# RippleNet: a Recurrent Neural Network for Sharp Wave Ripple (SPW-R) Detection

**DOI:** 10.1007/s12021-020-09496-2

**Published:** 2021-01-04

**Authors:** Espen Hagen, Anna R. Chambers, Gaute T. Einevoll, Klas H. Pettersen, Rune Enger, Alexander J. Stasik

**Affiliations:** 1 Faculty of Science and Technology, Norwegian University of Life Sciences, Ås, Norway; 2 Department of Physics, Faculty of Mathematics and Natural Sciences, University of Oslo, Oslo, Norway; 3 Division of Physiology, Department of Molecular Medicine, Institute of Basic Medical Sciences, Faculty of Medicine, University of Oslo, Oslo, Norway; 4 NORA - Norwegian Artificial Intelligence Research Consortium, Faculty of Mathematics and Natural Sciences, University of Oslo, Oslo, Norway; 5 Division of Anatomy, Department of Molecular Medicine, Institute of Basic Medical Sciences, Faculty of Medicine, University of Oslo, Oslo, Norway

**Keywords:** Machine learning, Deep learning, Recurrent neural networks, Neuroscience, Sharp wave ripples (SPW-R), Hippocampus CA1

## Abstract

Hippocampal sharp wave ripples (SPW-R) have been identified as key bio-markers of important brain functions such as memory consolidation and decision making. Understanding their underlying mechanisms in healthy and pathological brain function and behaviour rely on accurate SPW-R detection. In this multidisciplinary study, we propose a novel, self-improving artificial intelligence (AI) detection method in the form of deep Recurrent Neural Networks (RNN) with Long Short-Term memory (LSTM) layers that can learn features of SPW-R events from raw, labeled input data. The approach contrasts conventional routines that typically relies on hand-crafted, heuristic feature extraction and often laborious manual curation. The algorithm is trained using supervised learning on hand-curated data sets with SPW-R events obtained under controlled conditions. The input to the algorithm is the local field potential (LFP), the low-frequency part of extracellularly recorded electric potentials from the CA1 region of the hippocampus. Its output predictions can be interpreted as time-varying probabilities of SPW-R events for the duration of the inputs. A simple thresholding applied to the output probabilities is found to identify times of SPW-R events with high precision. The non-causal, or bidirectional variant of the proposed algorithm demonstrates consistently better accuracy compared to the causal, or unidirectional counterpart. Reference implementations of the algorithm, named ‘RippleNet’, are open source, freely available, and implemented using a common open-source framework for neural networks (tensorflow.keras) and can be easily incorporated into existing data analysis workflows for processing experimental data.

## Introduction

Transient and persistent oscillations or rhythms are ubiquitous in recordings of the brain’s activity (Buzsáki 2004; Wang 2010). Electric recordings of neural activity are indispensable tools in order to understand specific brain functions, but the measurements are commonly subject to poor signal-to-noise ratios due to noise and artefacts, especially *in vivo*. For accurate detection of specific neural signatures, improved methods for use in experimental and clinical work therefore need to be developed.

Sharp wave ripples (SPW-R) are brief, highly synchronous, fast oscillations observed in the CA1 region of the hippocampus of mammals. SPW-Rs arise in sleep and resting states, and originate in the hippocampal CA3 region (‘sharp waves’) and can elicit fast oscillations in the hippocampal CA1 region (‘ripples’). Features of SPW-Rs are highly preserved across species, and are linked to mechanisms that play important roles in memory function such as memory consolidation and recall of episodic memory. Excitatory output from the CA1 region during ripples encodes sequences of neuronal activation of awake experiences, that reaches wide areas of the cortex as well as subcortical nuclei. For a comprehensive review on SPW-Rs, their origin and function, see for example Buzsáki (2015).

**Fig. 1:**
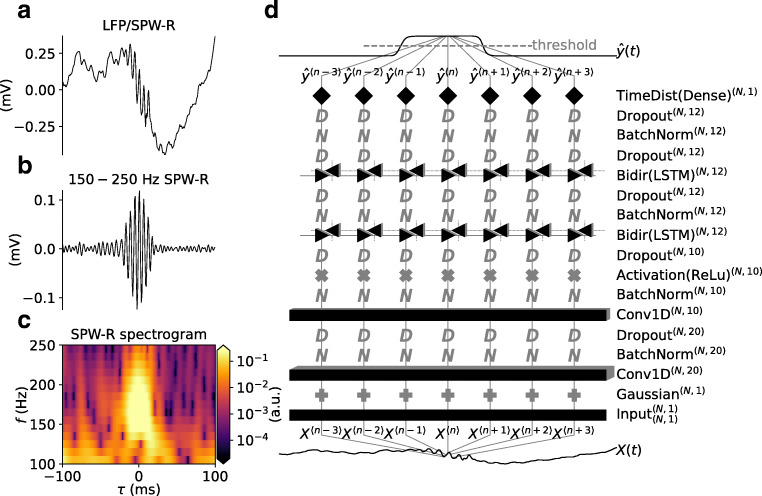
Example of a single detected SPW-R event and application of RippleNet to LFP data. **a** Raw LFP data with an SPW-R event. **b** Band-pass filtered LFP signal (150–250 Hz). **c** LFP spectrogram. **d** Illustration of a bidirectional RippleNet instance and its application to LFP signal *X*(*t*) (bottom trace) for predicting time-varying probabilities of SPW-R events $\hat {y}(t)$ (top trace). The subscript and superscripts annotating each layer denotes input and output dimensions respectively for an input sequence of length *N*

SPW-Rs occur as large amplitude oscillatory deflections of the local field potential (LFP) signal, the low-frequency ($\lesssim 300$ Hz) part of extracellularly recorded electric potentials measured in neural tissue. The SPW-R oscillations are observed above the cortical *γ*-band frequencies (30 − 90 Hz) (da Silva 2013) of the LFP, and lie between 160 − 180Hz in mice (Buzsáki et al. 2013, 2003), and between 130 − 160 Hz in rats (Buzsáki et al. 2013, 1992; O’Keefe and Nade 1978). Features of one such example SPW-R event are illustrated in Fig. 1. The wide-band LFP (Fig. 1a) contains a transient oscillation in the 150-250 Hz range (Fig. 1b), which is evident in the time-frequency resolved LFP spectrogram (Fig. 1c). The filtered signal and spectrogram is typically used by the experimentalist for SPW-R detection and manual verification.

Understanding the varying mechanisms linked to SPW-R events remains a highly active field of research, and mandates the continued development of precise and automated detection algorithms of these heterogeneous events. Their detection is further complicated by that the fractions of neurons in hippocampal regions CA1 and CA3 which are active during different SPW-R events vary greatly, and the number of small and medium-sized events outnumber large, highly synchronous events (Csicsvari et al. 1999a; Buzsáki 2015). Hence, the resulting distributions of SPW-R power are skewed as the synchrony between neurons throughout the network (i.e., correlations) greatly affects the LFP power in the SPW-R band (Csicsvari et al. 2000; Schomburg et al. 2012; Buzsáki 2015; Hagen et al. 2016). Consequently, defining a fixed threshold for SPW-R detection based on e.g., the power or envelope of the LFP in a chosen frequency band (Csicsvari et al. 1999a, 1999b; Einevoll et al. 2013; Ramirez-Villegas 2015; Norman et al. 2019; Tingley and Buzsáki 2020) remains heuristic. Detection methods may however incorporate adaptive thresholding (Fritsch et al. 1999; Jadhav et al. 2012).

Recent years have seen a surge in different supervised and unsupervised learning algorithms, propelled by hardware acceleration, better and larger training datasets, the advent of deep convolutional neural networks (CNN) in image classification and segmentation tasks (see e.g., LeCun et al. 2015; Rawat and Wang 2017) and high level software frameworks for neural networks (e.g., Tensorflow, Abadi et al. 2015; Keras, Chollet et al. 2015; Gulli and Pal 2017; PyTorch, Paszke et al. 2017). Deep CNNs are, however, not yet as commonplace for time series classification tasks (Fawaz et al. 2019). Unlike traditional neural networks (NNs) and CNNs which typically employ a feed-forward hierarchical propagation of activation across layers, *recurrent neural networks* (RNN) have feedback connections, and is suitable for sequential data such as speech and written text. One architecture of RNNs is Long Short-Term Memory (LSTM) RNNs (Hochreiter and Schmidhuber 1997), capable of classifying, processing and predicting events in time-series data, even in the presence of lags of unknown duration. In speech recognition, deep RNNs with multiple stacked LSTM layers have been successful in classifying phonemes (Graves et al. 2013). Bidirectional LSTM RNNs were also found to improve classification performance over unidirectional LSTM RNNs, which can only account for past context (Graves et al. 2013). The present context of SPW-R detection is analogous and amounts to recognition of a single phoneme or word in a temporal sequential signal such as sound.

Here, motivated by RNNs shown to be successful on speech-recognition tasks, we propose the utilization of LSTM-based RNNs for the automated detection of SPW-R events in continuous LFP data. As illustrated in Fig. 1d, our open-source implementation, RippleNet, is built with a combination of convolutional, (bidirectional) LSTM and dense layers with non-linear activation functions. Notably, RippleNet accepts raw, single-channel LFP traces (bottom trace) of arbitrary length as input. Thus the typical SPW-R detection steps of band-pass filtering the input LFP as well as manual feature extraction such as computing the signal envelope via the Hilbert transform or time-frequency resolved spectrograms are omitted. Using training data with labeled SPW-R events in real-world datasets from different sources we trained RippleNet instances to predict a continuous signal representing the time varying probability of SPW-R event in the input. A simple search of local peaks above a fixed threshold can then be applied with the output probabilities ($\hat {y}(t)$ in Fig. 1d).

Zuo et al. (2019) proposed one of few deep CNN based algorithms specifically designed for detection of high-frequency oscillations (HFO), that is, epileptogenic zone seizures in intracranial electroencephalogram (iEEG) recordings. While HFOs in epilepsy may be phenomenologically similar to hippocampal SPW-Rs, their origin is different. The RippleNet algorithm differs from Zuo et al. (2019) in that (1) explicit conversion of 1D input sequences with multiple rows into gray-scale images are avoided; (2) normalization of the input to zero mean and standard deviation to unity is not required; (3) input segments can be of arbitrary length (i.e., continuous) but segments within single batches have to be of the same length; (4) a fairly low number of parameters are trainable which may reduce overfitting; and (5) its outputs are continuous signals that represent the time varying probability of an SPW-R event at all time points of the inputs, in contrast to classifying whether or not a HFO class occurs in each fixed-size input segment. Similar to our approach, also Medvedev et al. (2019) used LSTM layers, but in a categorical classification task on fixed-size input spectrograms spanning eight frequency bands and three time steps. Our method uses the raw time series as input and does not rely on such preprocessing of the signal.

For the two main RippleNet variants we propose, one causal using unidirectional LSTM layers and a non-causal version using bidirectional LSTM layers, our main findings are that causal RippleNet instances can detect nearly the same number of actual SPW-R events as their non-causal variants (511 ± 8.24 vs. 527 ± 5.11), but at the cost of significantly higher error rate (84.3 ± 3.60 vs. 108 ± 1.75) on our validation data. Subsequent application of a candidate non-causal RippleNet instance to continuous test data demonstrate high temporal precision of event detection, high rate of true and false positives and low rate of false negatives, however, the false positive predictions overlap in features with true SPW-R events as judged by an expert. RippleNet also runs faster than real-time on typical CPUs, and even faster on graphical processing units (GPU).

## Methods

### Experimental Data

#### Mouse Data

**Fig. 2:**
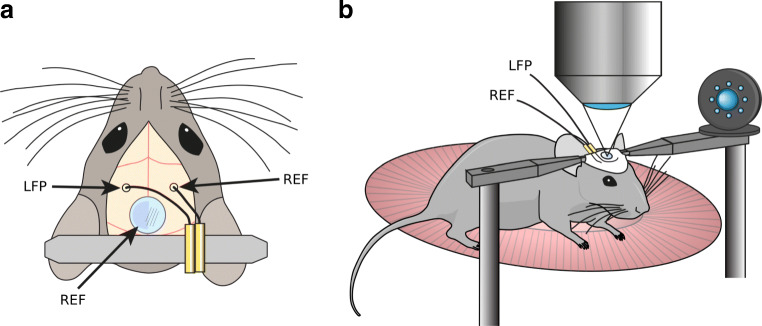
Experimental setup. A set of recordings used for this study were acquired concomitant to two-photon microscopy from head-fixed mice. **a** Mice were prepared with a single electrode in the the hippocampal CA1 region and a contralateral reference electrode, chronic glass window for two-photon microscopy and a head bar for head fixation. **b** LFP recordings were recorded concomitant to two-photon microscopy in head-fixed mice

Male and female mice (C57Bl/6J; Janvier labs) underwent LFP electrode implant surgery at approximately 10–14 weeks of age. All mice had previously been implanted 2–3 weeks earlier with a custom made titanium head bar glued to the skull and covered with a dental cement cap as illustrated in Fig. 2a. For electrode implant surgery, mice were anesthetized with isoflurane (3% induction, 1.5% maintenance, in pure oxygen) with body temperature maintained at 37^∘^C. Burr holes were drilled for the LFP electrode and reference electrode over the dorsal CA1 region of the hippocampus (A/P − 2 mm, M/L 2 mm) and contralateral primary somatosensory cortex (A/P − 0.5 mm, M/L 3 mm), respectively. Silver wire electrodes (0.125 mm diameter, insulated, GoodFellow) were lowered to a depth of 0.8 mm for dorsal CA1. The reference electrode was implanted at the brain surface. Mice were allowed to recover from isoflurane anesthesia while head fixed for at least 15 minutes, and electrode placement was confirmed by monitoring the LFP signal online. Electrodes were affixed to the head bar with cyanoacrylate glue and a thin layer of dental cement.

LFP recordings were band-pass filtered (0.1–1000 Hz) and amplified (1000×) with a DAM50 differential amplifier (World Precision Instruments Inc). Line noise was removed using a HumBug 50/60 Hz Noise Eliminator (Quest Scientific Inc). For experiments, mice were head fixed under a two-photon microscope objective after brief isoflurane anesthesia. They were given at least 15 minutes to recover from anesthesia before recordings were taken. In most cases, LFP recordings were performed concurrently with two-photon calcium imaging through a chronic cranial window over the retrosplenial cortex (Fig. 2a, b), in 10 minute sessions. During recordings, mice were able to walk freely on a disc (Fig. 2b) equipped with a rotary encoder to record locomotion, grooming and postural adjustments. Experiments were performed in the dark. LFP and rotary encoder signals were acquired at 20 kHz and downsampled to a final sampling frequency *f*_s_ = 2500 Hz in LabView (National Instruments). The LFP signals were saved in units of millivolts (mV).

The different animals, number of sessions, total recording durations and number of SPW-R events are listed in Table 1. All procedures were approved by the Norwegian Food Safety Authority (project: FOTS 19129). The experiments were performed in accordance with the Norwegian Animal Welfare Act and the European Convention for the Protection of Vertebrate Animals used for Experimental and Other Scientific Purposes.

**Table 1:** Summary of data acquisition, and extracted training, validation and test data

Animals and sessions			
Mouse ID	# sessions	Duration (s)	# SPW-R
4028	4	2412	425
4029	1	603	86
4030	4	2412	658
4031	2	1206	176
4046	4	2410	596
4104	1	603	181
4105	3	1809	231
4106	4	2412	382
4214	3	1812	491
4215	3	1812	440
6102	3	1812	467
6112	3	1812	376
Rat ID	# sessions	Duration (s)	# SPW-R
2	1	29283	4498
7	1	25575	3714
9	1	26434	3677
Extracted datasets			
**X**,**y**	*n*_SPW−R_: {4461, 4400} (mouse and rat)		
	shape: (*n*_SPW−R_, 1250, 1)		
**X**_train_,**y**_train_	*n*_train_: {4175, 4000}		
	shape: (*n*_train_, 1250, 1)		
**X**_val_,**y**_val_	*n*_val_: {200, 200}		
	shape: (*n*_val_, 1250, 1)		
**X**_test_,**y**_test_	*n*_test_: {1, 0}		
	shape: (*n*_test_, 753914, 1)		

##### SPW-R Detection Procedure

Pre-processing for manual SPW-R detection was performed using MATLAB (MATLAB 2018).^1^ The LFP signal was first band-pass filtered between 150 and 250 Hz using a digital filter filtfilt. The coefficients for the order 600 finite impulse response (FIR) filter were generated using the fir1 function. The band-pass filtered LFP was then used to compute the absolute of the Hilbert transform of the data. The output was smoothed by convolving with a 1052-point Gaussian filter with *σ* = 40 ms using gaussfilt (Conder 2020). The findpeaks function was used to find peaks which were 3 standard deviations above mean in a moving time window with duration 1s. A minimum peak width at half height of 15ms and a minimal peak distance of 25ms were required, calculated based on data reported in Axmacher et al. (2008), Davidson et al. (2009), and Caputi et al. (2012). Potential ripple locations where then manually inspected using the symmetric one second time window around it, based on the Hilbert transformation and the raw LFP signal.

#### Rat Data

To supplement the training and validation datasets containing SPW-R events that could be extracted from the in-house datas described above, we utilized publicly available datasets from the Buzsáki lab webshare^2^ (Petersen et al. 2018). The datas were obtained in the adult rat (Long Evans) in awake and sleep states using chronically implanted probes with a total of 96 or 128 channels (Tingley and Buzsáki 2018, 2020). The datasets were

- DT2/DT2_rPPC_rCCG_3612um_1360um_20160303 _160303_084915,
- DT7/20170324_576um_144um_170324_123932,
- DT9/20170509_468um_36um_170509_103451.

The LFP signal of contacts located in CA1 was extracted and converted to units of mV, along with the corresponding times and durations of labeled CA1 SPW-R events. SPW-R events were identified and labeled as described in Tingley and Buzsáki (2020). We here defined SPW-R event times as the mean of onset and offset times. All events in awake and sleep states were extracted. The sampling frequency of the LFP data was here *f*_s_ = 1250Hz.

The different animals, number of sessions, total recording durations and number of SPW-R events are summarized in Table 1.

### Data Preprocessing

The mouse LFP data were downsampled to a common sampling frequency *f*_s_ = 1250 Hz and temporal resolution *Δt* = 1/*f*_s_. For temporal downsampling we used the scipy.signal.decimate function with default parameters. The rat LFP datasets were used as is. For visualization, we extracted the band-pass filtered LFP *ϕ*_BP_(*t*) from the LFP *ϕ*(*t*) using 2nd-order Butterworth filter coefficients computed with critical frequencies *f*_c_ ∈{150,250} Hz. Filters were applied to *ϕ*(*t*) using a zero phase shift, forward-backward filter implementation. Filter coefficients were computed using scipy.signal.butter and applied with scipy.signal.filtfilt.

#### Wavelet Spectrograms

To compute spectrograms of LFP data *ϕ*(*t*) we relied on the complex Morlet transform with parameters *ω* = 6, scaling factor *s* = 1 and lengths *M*_*f*_ = 2*sf*_s_*ω*/*f* for fundamental frequencies *f* ∈{100,110…,240,250} Hz. The numbers *M*_*f*_ were rounded down to the nearest integer. The set of discrete wavelet coefficients for each frequency *f* were computed using the function scipy.signal.morlet as

1 $$ \begin{array}{@{}rcl@{}} \varPsi_{f} &=& \pi^{-0.25}\mathrm{e}^{-0.5 x^{2}} (\mathrm{e}^{j \omega x} - \mathrm{e}^{-0.5 \omega^{2} }) ~\text{with} \end{array} $$

2 $$ \begin{array}{@{}rcl@{}} x &\in& \left\{-2 \pi s, -2 \pi s \left( 1-\frac{2}{M_{f}} \right), \ldots, 2 \pi s \right\}. \end{array} $$

Each row of the spectrograms *S*(*t*,*f*) = [*S*_*f*_(*t*)] were then computed for all frequencies in *f* as

3 $$ S_{f}(t) = |(\phi * \varPsi_{f})(t)|^{2}, $$

where the asterisk denotes a convolution. We used the discrete 1D implementation by scipy.signal. convolution in ‘full’ mode. To visualize the spectrograms, we employed a log-linear matplotlib. cm.inferno color map, with lower and upper limits determined as $\exp (c)$, where *c* is the 1st and 99th percentiles of $\log (S)$, respectively.

#### Training, Validation and Test Data

##### Input Data

We chose to use the raw single-channel LFP data segments as input to the neural network algorithm, that is, by defining *X*(*t*) = [*ϕ*(*t*)]. For reasons related to the RNN implementation we defined each segment *X*(*t*) as shape (*n*_timesteps_,1) arrays, even if we here work with single-channel LFP data.

##### One-Hot Encoding of SPW-R Events

The train of *n* labeled times *t*^〈*i*〉^ of the SPW-R events in each continuous LFP data segment can be expressed mathematically as

4 $$ T(t) = \sum\limits_{i=1}^{n} \delta(t-t^{\langle i \rangle}), $$

where *δ*(⋅) denotes the dirac delta distribution, and *i* the index of the event in a session. We then assumed that each SPW-R has a typical duration *D* = 50 ms on the interval [*t*^〈*i*〉^− *D*/2,*t*^〈*i*〉^ + *D*/2). A binary ‘one-hot’ encoding vector for the SPW-R events *y*(*t*) was then computed as

5 $$ \begin{array}{@{}rcl@{}} y(t) &=& \min\left( \varphi(t), 1\right), \text{where} \end{array} $$

6 $$ \begin{array}{@{}rcl@{}} \varphi(t) \!\!&=&\! \!\left( \left( \theta(t - t^{\langle i \rangle} + D/2) - \theta(t - t^{\langle i \rangle}-D/2) \right) * T \right)(t), \end{array} $$

for the entire duration of each LFP segment. Here *𝜃*(⋅) denotes the Heaviside step function. The vector *y*(*t*) can be interpreted as the time-varying, binary probability *p* ∈ [0,1] of an SPW-R occurring at any given time *t*.

##### Datasets

As the SPW-R occurrence in the data was *sparse* (that is, *y*(*t*) = 0 for most *t*), training the neural network on entire data segments of different durations is impractical. A likely training outcome is predicting $\hat {y}(t)=0$ for all times *t* of the input. For each labeled SPW-R event we therefore extracted temporal segments of duration *T*_sample_ = 1000 ms from *X*(*t*) and *y*(*t*), that is, on the interval $[t^{\langle j \rangle } - T_{\text {offset}}^{\langle j \rangle } - T_{\text {sample}}/2, t^{\langle j \rangle } - T_{\text {offset}}^{\langle j \rangle } + T_{\text {sample}}/2)$. The offsets $T_{\text {offset}}^{\langle j \rangle } \in [-(T_{\text {sample}} - 3D)/2, (T_{\text {sample}} - 3D)/2)$ were randomly drawn for each event. The superscript 〈*j*〉 here denotes a sample indexed by *j* from any LFP recording session.

For the total number *n*_SPW−R_ of SPW-R samples across all animals and sessions, the shapes of the combined input and output dataset matrices **X** and **y** for training, validation and testing were both (*n*_SPW−R_,*T*_sample_/*Δt*,1).

**Table 2:**
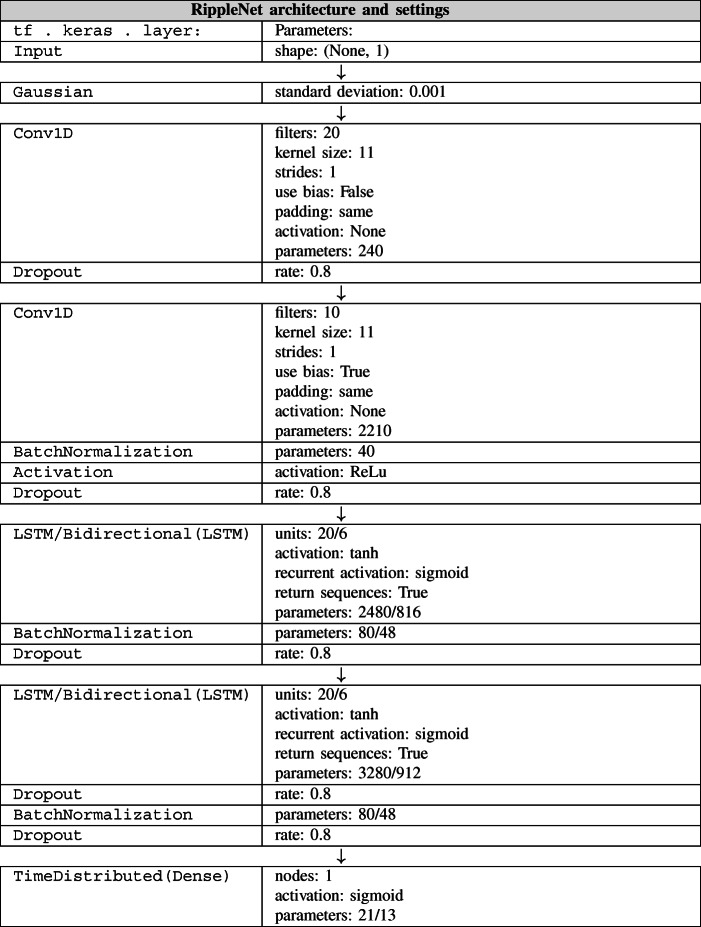
RippleNet neural network structure and parameters for both unidirectional and bidirectional variants

All data entries except for a hold-out set were randomly reordered along their first axis, and then split into 2 separate file sets for validation and training, each of sizes summarized in Table 1. The validation set was used to monitor loss during training and quantification of performance as detailed below. The hold-out test set constructed from the entire session of one animal (mouse 4029) was only utilized for final evaluation of the RNN after training and validation. For visualization purposes we also stored the corresponding segments of band-pass filtered LFP ($\phi _{\text {BP}}^{\langle j \rangle }(t)$) and spectrograms (*S*^〈*j*〉^(*t*,*f*)) for every labeled event.

In an effort to balance the set of features that can be learned by RippleNet from datas obtained mice and rat, we extracted a similar count of SPW-R events for training and validation from the rat data as for the mouse data.

### RippleNet Implementations

The causal and non-causal RippleNet implementations, summarized schematically and with parameters in Table 2, consist of a Gaussian noise layer applied to the input (Matsuoka 1992), then one 1D convolutional layer (LeCun et al. 1990) followed by a dropout layer (Srivastava et al. 2014), followed by another 1D convolutional layer followed by batch-normalization (Ioffe and Szegedy 2015), rectified-linear (ReLu) activation (Nair and Hinton 2010) and dropout layers. The output of the last convolutional layer are consecutively fed to the first (bidirectional) LSTM layer (Hochreiter and Schmidhuber 1997) followed by batch-normalization and dropout. The final (bidirectional) LSTM layer is followed by a dropout, batch-normalization and a final dropout layer. Bidirectional layers are optionally applied using a wrapper function. The output is governed by a time-distributed layer wrapping a dense layer (Rosenblatt 1958) with sigmoid activation, which facilitates application of the dense layer to every temporal slice of the input. Hence, the output is a matrix with the same dimension as the input.

The Gaussian noise layer and dropout layers are only active during training in order to prevent overfitting of the training set, and inactive during validation and testing. The kernel weights of the convolutional, dense and LSTM layers are initialized with the Glorot uniform initializer. Recurrent connections in LSTM layers are initialized using the Orthogonal initializer. For optimization we chose the Adam algorithm which implements an adaptive stochastic gradient descent method (Kingma and Ba 2014). The settings for model compilation, optimization algorithm and model fitting are summarized in Table 3.

**Table 3:** Summary of settings for model compilation, optimization and fitting of training data set

Model and optimizer settings	
tf.keras method:	Parameters:
Model.compile	loss: binary_crossentropy
	optimizer: tf.keras.optimizers.Adam
	metrics: mse
optimizers.Adam	lr (learning rate): 0.005/0.01
	beta_1: 0.9
	beta_2: 0.999
	epsilon: 1e-07
Model.fit	**X**_**train**_: shape (*n*_train_, 1250, 1) array
	**y**_**train**_: shape (*n*_train_, 1250, 1) array
	batch size: 20
	epochs: 50
	**X**_**val**_: shape (*n*_val_, 1250, 1) array
	**y**_**val**_: shape (*n*_val_, 1250, 1) array

Layer dimensions were hand tuned, with the aim of reducing the amount of trainable parameters and reducing evaluation times and overall training times, while maintaining achievable loss *J* and *MSE* reasonably low.

For training the RNN we used the binary cross entropy loss function (tf.keras.losses.Binary Crossentropy)

7 $$ J = -\frac{1}{N} \sum\limits_{n=1}^{N} \left[y(n) \log \hat{y}(n) + \left( 1 - y(n)\right) \log\left( 1-\hat{y}(n)\right) \right], $$

where *N* = *T*_sample_/*Δt* is the number of temporal samples in the label array *y* and RNN prediction $\hat {y}$. To monitor training and validation performance of the RNN we used the mean squared error

8 $$ MSE = \frac{1}{N} \sum\limits_{n=1}^{N} \left( y(n) - \hat{y}(n)\right)^{2}. $$

For 3-fold cross-validation of different causal and non-causal RippleNet variants, each instance is initialized using different random seeds affecting pseudo-random number generation for initializers, Gaussian noise and dropout layers and optimization. We set the environment variables PYTHONHASHSEED=<seed value> and TF_DETERMINISTIC_OPS=‘1'. These steps ensure *methods reproducibility* (Plesser 2018) and deterministic training results (i.e., network weight updates) on similar GPU hardware and software versions.

### Data Analysis

#### Thresholding of RippleNet Predictions

The output $\hat {y}^{\langle j \rangle }(t) \in (0, 1)$ of RippleNet is a discrete signal of same temporal duration and resolution as an input segment *X*^〈*j*〉^(*t*). The signal $\hat {y}^{\langle j \rangle }(t)$ can be interpreted as the time-varying probability of an SPW-R ripple event. To extract time points $\hat {t}_{\mathrm {SPW-R}}$ of candidate ripple events, we ran the peak-finding algorithm implemented by scipy.signal.find_peaks using an initial threshold of 0.5, minimum peak inter-distance of 50ms (same as *D*) and peak width of 20ms. Other parameters were left at their default values. Further analyses of SPW-R detection performance were conducted in a discrete grid search by varying the threshold between 0.1 and 0.95 in increments of 0.5 and peak width between 0 and 50 ms in increments of 5 ms, assessing the influence on the metrics defined next.

#### Quantification of True and False Detections

On the validation and test data sets, we counted a true positive (*TP*) for the predicted time $\hat {t}_{\mathrm {SPW-R}}$ of an SPW-R event if $y(\hat {t}_{\mathrm {SPW-R}})=1$, false positive (*FP*) if $y(\hat {t}_{\mathrm {SPW-R}})=0$ and false negative (*FN*) if no peaks above threshold in $\hat {y}(t)$ were found in time intervals where *y*(*t*) = 1. $\hat {y}(t)$ can be above threshold if FP predictions occur next to labeled SPW-R events and result in FN counts. Negative samples, where *y*(*t*) = 0 for all times spanned by the LFP samples, were not included in any of the training, validation or test sets. Hence, evaluation of true negative (*TN*) predictions were not performed. Note, however, that by construction, each sample *y*^〈*j*〉^(*t*) was equal to zero up to 95 *%* of the time spanned by the sample, and that more than one SPW-R event may exist in each segment.

#### Precision, Recall and *F*_1_ Metrics

The following quantification metrics of SPW-R detection performance are used:

9 $$ \begin{array}{@{}rcl@{}} \textit{Precision} &=& \frac{TP}{TP + FP}, \end{array} $$

10 $$ \begin{array}{@{}rcl@{}} \textit{Recall} &=& \frac{TP}{TP + FN}, \end{array} $$

11 $$ \begin{array}{@{}rcl@{}} F_{1} &=& 2 \cdot \frac{\textit{Precision} \cdot \textit{Recall}}{\textit{Precision} + \textit{Recall}}. \end{array} $$

*Recall* is sometimes referred to as *True Positive Rate (TPR)* and *Sensitivity* in the literature (e.g., by Zuo et al. 2019). *Precision* is also known as *Positive Predictive Value (PPV).* The *F*_1_ score represents the harmonic mean of *Precision* and *Recall*. These metrics are all defined on the interval [0,1], with 1 implying perfect performance.

#### Temporal Correlation Analysis

To quantify the temporal agreement with labeled and predicted SPW-R event times, we compute the cross-correlation coefficients between predicted ripple event times $\hat {t}^{\langle j \rangle }$ and labeled event times *t*^〈*j*〉^ as function of time lag *τ* as (Eggermont 2010, Eq. 5.10):

12 $$ \begin{array}{@{}rcl@{}} \rho_{\upsilon\hat{\upsilon}}(\tau)\! &=& \!\left( R_{\upsilon\hat{\upsilon}}(\tau) - \frac{N_{\upsilon} N_{\hat{\upsilon}}}{N} \right) \left( \left( 1 - \frac{N_{\upsilon}}{N} \right) \left( 1 - \frac{N_{\hat{\upsilon}}}{N} \right)\right)^{-\frac{1}{2}}, \text{where} \\ \end{array} $$

13 $$ \begin{array}{@{}rcl@{}} R_{\upsilon\hat{\upsilon}}(k) &=& \frac{1}{N} \sum\limits_{n=1}^{N} \upsilon(n) \hat{\upsilon}(n+k). \end{array} $$

Here, *υ* and $\hat {\upsilon }$ are the time binned *N*_*υ*_ and $N_{\hat {\upsilon }}$ times of labeled and predicted SPW-R events using a bin width *Δ* = 2 ms, where $N = N_{\upsilon } + N_{\hat {\upsilon }}$.

#### Quantification of Signal Energy

To quantify ‘strengths’ of ripples in the band-pass filtered LFP, we compute the signal energy (not to be confused with energy in physics) as

14 $$ E_{s}^{\langle j \rangle} = \sum\limits_{n=1}^{N} |\phi_{\text{BP}}^{\langle j \rangle}(n)|^{2},  $$

where *N* = 2*τ*/*Δt* and *τ* ∈ [− 100,100] ms denotes time relative to the SPW-R event time.

### Technical Summary

The Python-based preprocessing and data extraction steps used Python^3^ (v3.6.10), jupyter-notebook^4^ (v6.0.3), numpy^5^ (v1.18.1, van der Walt et al. 2011), scipy^6^ (v1.4.1, Vurtanen et al. 2020), h5py^7^ (v2.10.0, Collette et al. 2019), matplotlib^8^ (v3.2.1, Hunter 2007), pandas^9^ (v1.0.3, McKinney 2010) with the Anaconda Python Distribution^10^ (v4.8.3) running on a 13-inch 2016 Macbook Pro with macOS Mojave (v10.14.6).

The main training, analysis and visualization of performance of RippleNet was implemented and executed using Python (v3.6.9), jupyter-notebook (v5.2.2), numpy (v1.18.2), scipy (v1.4.1), h5py (v2.10.0), matplotlib (v3.2.1), seaborn^11^ (v0.10.1, Waskom et al. 2020), pandas (v1.0.3) and tensorflow^12^ (v2.1.0, Abadi et al. 2015) running on the Google Colaboratory portal^13^ using GPU hardware acceleration (using single Nvidia K80s, T4s, P4s or P100s cards).

## Results

We here present our main findings and analysis of RippleNet, a set of automated, trainable recurrent neural network algorithms for detecting SPW-R events in single-channel LFP recordings. For training and evaluation of RippleNet instances, the full dataset (**X**,**y**) with LFP segments and labels is split into separate training, validation and test sets with dimensions detailed in Table 1.

### Experimental Datasets for Training and Validation

**Fig. 3:**
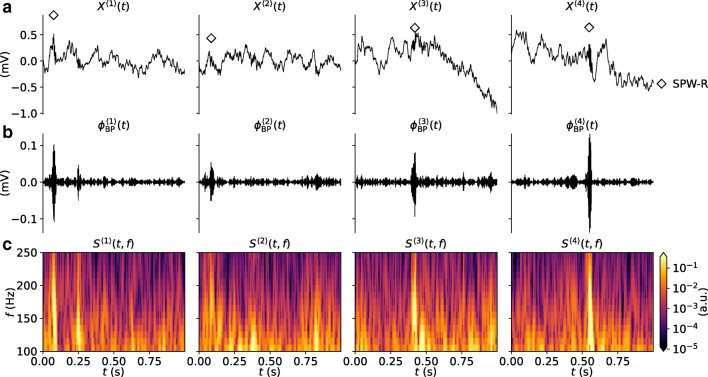
Snapshots of experimental data. **a** Samples of raw LFP traces (*X*^〈1〉^(*t*),*X*^〈2〉^(*t*),…) with at least one labeled SPW-R event. The *diamonds* mark the times of the labeled events. Each column corresponds to samples *j* from the validation dataset. **b** Band-pass filtered LFP traces $\phi _{\text {BP}}^{\langle j \rangle }(t)$. **c** Wavelet spectrograms *S*^〈*j*〉^(*t*,*f*) computed from the LFP traces

Brain signals such as the LFP are characterized by low-frequency fluctuations, with spurious oscillatory events that may occur in different parts of the frequency spectrum. A few 1s samples of mouse hippocampus CA1 LFP from our validation data $X^{\langle j \rangle }(t) \in \mathbf {X}_{\text {val}}$ are shown in Fig. 3a. Each sample contains at least one labeled SPW-R event verified by a domain expert at times marked by the diamond symbols. The SPW-R events identified using a conventional method involving manual steps (cf. “Methods”), are hardly discernible by eye. They stand out, however, in the corresponding band-pass filtered LFP signals $\phi _{\text {BP}}^{\langle j \rangle }(t)$ (Fig. 3b) and in the time-frequency resolved spectrograms *S*^〈*j*〉^(*t*,*f*) (Fig. 3c). Individual samples may also include potential SPW-R events that were not labeled. Events may have amplitudes of $\sim 0.1$ mV in the filtered signal. Their durations are also short ($\lesssim 100$ ms).

### Training and Validation of RippleNet Variants

We next continue with 3-fold cross validation of two main variants of RippleNet during and after training, that are either unidirectional (causal) or bidirectional (non-causal). The RippleNet model instances are initialized in each trial with different random seeds affecting initial weights, parameters, Gaussian noise, dropout and subsequent weight updates during training. The counts of trainable parameters is kept about a factor two higher for the unidirectional variant (cf. the full descriptions of each variant in Table 2). The computational load during training was approximately a factor two higher for the bidirectional variant. We observed $\sim $90 and $\sim $170 ms/step on Tesla P4 GPUs during training, respectively. One step corresponds to one batch of 20 samples.

**Fig. 4:**
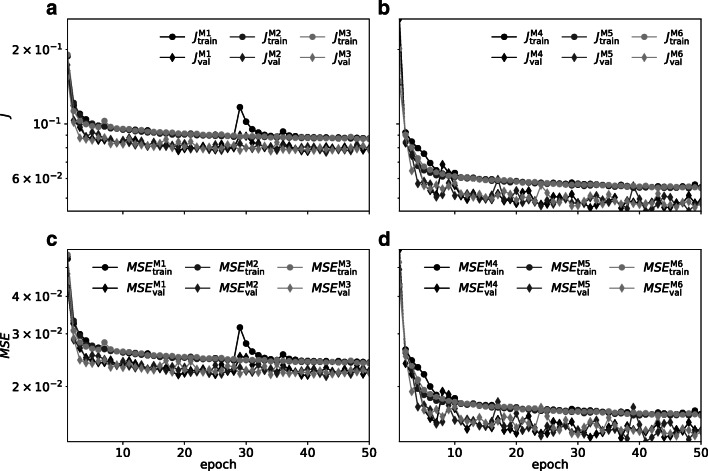
**a** Training and validation loss *J* as function of training epoch for for unidirectional RippleNet variants. Each instance M1-3 are instantiated using different random seeds. **b** same as panel (**a**), but for the non-causal bidirectional variant. **c**, **d** Training and validation *MSE* as function of training epoch

The training and validation loss *J* and *MSE* as function of training epoch are shown in Fig. 4a, b and c, d, respectively. Model instances M1-3 in Fig. 4a, c are of the unidirectional variant, while models M4-6 in Fig. 4b and d are bidirectional. Instances of each variant, except M1, display similar and stable trajectories during training. Validation loss *J* and *MSE* are as expected inherently more variable across epochs, due to the smaller number of validation samples. Both in terms of loss *J* and *MSE* the bidirectional instances performs consistently better than the unidirectional instances after just a few training epochs. Validation loss *J* and *MSE* are reduced compared to training outcome as noise and dropout layers are inactive during validation. The different trajectories indicate no signs of over-fitting either to the training or validation sets. For further analysis we therefore chose trained network instances after the final 50th training epoch.

#### Validation Set Performance

**Fig. 5:**
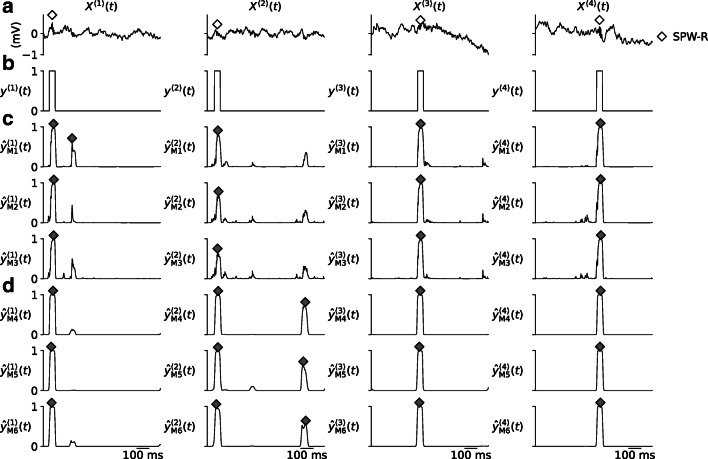
Comparison of RippleNet predictions on samples from the validation set. Each column corresponds to different input LFP samples *X*^〈*j*〉^ shown at the *top*. **a**) Input LFP samples *X*^〈*j*〉^. The *diamonds* mark the times of labeled SPW-R events. **b** One-hot encoded label vectors *y*^〈*j*〉^(*t*). **c** Predictions $\hat {y}^{\langle j \rangle }(t)$ made by the different instances of the unidirectional RippleNet variant. SPW-R events found by the peak-finding algorithm are marked with *diamond markers*. **d** Same as panel (**c**), but for the bidirectional RippleNet variant

Training and validation losses *J* and *MSE* only provide an indication of the ability to detect SPW-R events using the different model instances. First, in Fig. 5 we visually compare a subset of predictions $\hat {y}^{\langle j \rangle }(t)$ on LFP samples from a validation set ($X^{\langle j \rangle }(t) \in \mathbf {X}_{\text {val}}(t)$), to one-hot encoded SPW-R events *y*^〈*j*〉^(*t*) (see “Methods”). Here, all model instances produce predictions (Fig. 5c–d) with responses above the detection threshold for labeled events, but spurious threshold crossings may occur elsewhere.

The non-causal bidirectional RippleNet instances (models M4-6, Fig. 5d) produce output with notably less spurious fluctuations below threshold, when compared to the causal instances (M1-3, Fig. 5c). These spurious fluctuations reflect the fact that signal power in the expected frequency range of SPW-R events do not vanish due to other ongoing neural processes, measurement noise etc. The bidirectional models do an overall better job at predicting the boxcar shapes of the one-hot encoded SPW-R events in Fig. 5b, owing to the fact that the full input time series are factored into their predictions.

We next quantify the different model instances’ performance in terms of counts of true positives (TP), false positives (FP) and false negative (FN) on the full validation set. Summarized in Table 4, trained instances of each variant (uni- vs. bidirectional RippleNets) show similar numbers of TP events, 495 ± 17.5 vs. 506 ± 13.9, using the initial settings for the peak-finding algorithm when applied to the predictions $\hat {y}^{\langle j \rangle }(t)$. However, total error counts (FP plus FN counts) are consistently higher for the unidirectional RippleNets compared to their bidirectional counterparts (131 ± 7.02 vs. 86.7 ± 7.09).

Summarized in Table 4 we also compute the corresponding measures of performance from the TP, FP and FN counts: *Precision*, the ratio between TP predictions and total number of predictions; *Recall*, the ratio between TP predictions and the sum of TP and FN predictions; and *F*_1_, the harmonic mean between *Precision* and *Recall*. Bidirectional instances show on average higher *Precision* than unidirectional instances (0.935 ± 0.012 vs. 0.881 ± 0.018), while *Recall* values are similar (0.886 ± 0.031 vs. 0.908 ± 0.024). Bidirectional models which show an overall better performance in terms of TP, FP and TN counts with *F*_1_ scores of 0.921 ± 0.008, above the corresponding values for the unidirectional instances (0.883 ± 0.008).

#### Effect of Detection Threshold and Width Parameters

**Table 4:** TP, FP, TN counts and performance metrics for RippleNet models on validation data

Model performance summary								
Variant	Model	*TP*	*FP*	*FN*	*FP* + *FN*	*Prec*.	*Recall*	*F*_1_
unidir.	1	512	83	47	130	0.861	0.916	0.887
	2	477	57	81	138	0.893	0.855	0.874
	3	495	61	63	124	0.89	0.887	0.889
unidir.	mean	495	67	63.7	131	0.881	0.886	0.883
	st.dev	17.5	14	17	7.02	0.018	0.031	0.008
bidir.	4	522	43	36	79	0.924	0.935	0.93
	5	499	36	57	93	0.933	0.897	0.915
	6	497	28	60	88	0.947	0.892	0.919
bidir.	mean	506	35.7	51	86.7	0.935	0.908	0.921
	st.dev	13.9	7.51	13.1	7.09	0.012	0.024	0.008

**Fig. 6:**
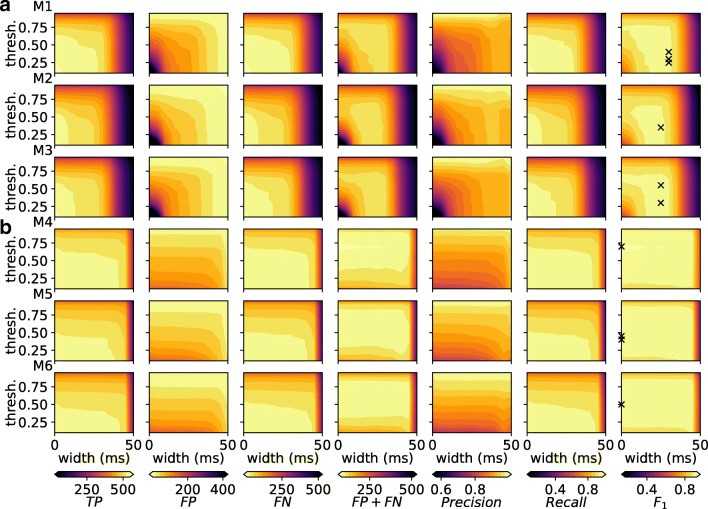
Effect of varying threshold and width parameters for the peak finding algorithm on counts of TP, FP and FN events in the validation dataset and derived metrics for different RippleNet instances. **a** Each row corresponds to different model instances of the unidirectional RippleNet variant. The columns correspond to different metrics. *Colorbars* are shared among panels in each column. The *cross hatches* in the last *F*_1_ column correspond to parameter combinations maximizing the *F*_1_ score as summarized in Table 5. **b** Same as panels in (**a**) but for instances of the bidirectional RippleNet variant

The above analysis assumes fixed hyper-parameters for the peak-finding algorithm (cf. “Methods”) applied to the predictions by RippleNet instances on the validation set. These hyper-parameters include threshold, minimal peak interdistance and width (in units of time steps of size *Δt*). We next hypothesize that the total error counts (FP+FN) can be minimized and correct prediction counts (TP) can be maximized using a hyper-parameter grid search, and choose to optimize thresholds and widths for each network with respect to the *F*_1_-score. We keep the minimal peak interdistance the same as the boxcar filter width used to construct *y*(*t*). Summarized in Fig. 6, the TP and FP counts for each model instance increased when lowering the threshold and width. FN counts increase for high threshold values and widths. Bidirectional model instances (M4-6 in Fig. 6b) are less affected by the width setting compared to instances of the unidirectional variant (M1-3 in Fig. 6a). The different instances display different ‘sweet spots’ in terms of total number or errors (FP+FN). These counts are reflected in the calculated *Precision* and *Recall* values. The *F*_1_ space show for some instances multiple local maxima as summarized in Table 5. Here model 4 (M4) has the overall best performance, both in terms of least amounts of errors and highest *F*_1_ score. For further analysis and later application to a hidden test set we therefore choose that model, with detection threshold 0.7 and peak width of 0 time steps. In passing, we note that the other two bidirectional RippleNet instances achieve nearly similar levels of performance, while unidirectional instances have higher error counts as reflected in the *F*_1_ values (0.926 ± 0.003 vs. 0.905 ± 0.0).

**Table 5:** TP, FP, TN counts and performance metrics for different RippleNet instances on validation datasets, using threshold settings optimized with respect to maximizing *F*_1_

Optimized models performance summary										
Variant	Model	thresh.	width	*TP*	*FP*	*FN*	*FP* + *FN*	*Prec*.	*Recall*	*F*_1_
unidir.	1	0.25	37.5	518	71	38	109	0.879	0.932	0.905
	1	0.3	37.5	518	71	38	109	0.879	0.932	0.905
	1	0.4	37.5	517	70	39	109	0.881	0.93	0.905
	2	0.35	31.2	498	47	58	105	0.914	0.896	0.905
	3	0.3	31.2	512	64	44	108	0.889	0.921	0.905
	3	0.55	31.2	505	55	51	106	0.902	0.908	0.905
unidir.	mean	0.358	34.4	511	63	44.7	108	0.891	0.92	0.905
	st.dev	0.107	3.42	8.24	9.98	8.24	1.75	0.014	0.015	0
bidir.	4	0.7	0	522	43	36	79	0.924	0.935	0.93
	5	0.4	0	534	63	24	87	0.894	0.957	0.925
	5	0.45	0	527	56	30	86	0.904	0.946	0.925
	6	0.5	0	525	53	32	85	0.908	0.943	0.925
bidir.	mean	0.512	0	527	53.8	30.5	84.2	0.907	0.945	0.926
	st.dev	0.131	0	5.1	8.3	5	3.59	0.013	0.009	0.003

#### False (FP & FN) Predictions

**Fig. 7:**
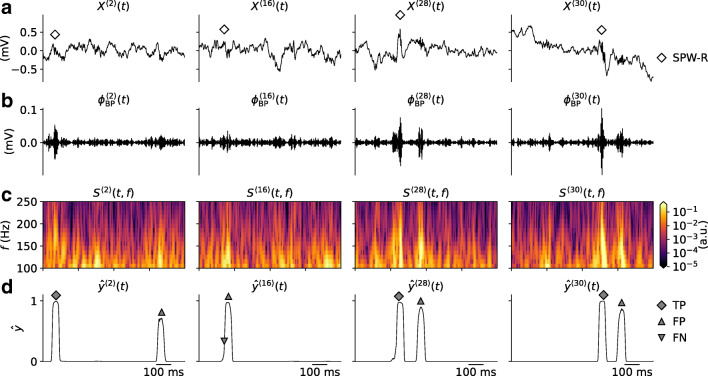
Examples of validation samples *j* resulting in at least one FP prediction per sample. FN predictions may also occur. Columns show **a** input sequences with times of labeled SPW-R events denoted by diamond markers, **b** band-pass filtered LFP, **c** spectrograms and **d** predictions with detected events. The *diamond* and *upward/downward pointing triangle* markers denote times of TP, FP and FN events, respectively

**Fig. 8:**
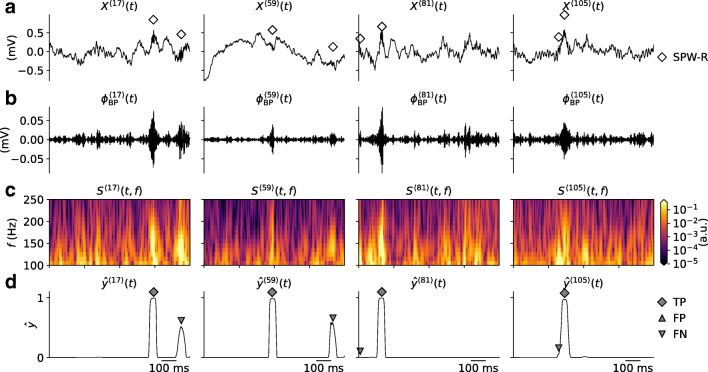
Same as Fig. 7, but showing a set of samples with at least one FN prediction per sample

Having assessed the best performing bidirectional RippleNet model instance and corresponding combination of width and threshold parameters on the validation set (Table 5), we next inspect features of FP and FN predictions on the validation dataset. This step can expose latent issues with the data and/or the predictions made by the trained network. LFP samples resulting in FP and FN predictions are illustrated in Figs. 7–8, respectively. From this subset of samples, FP predictions appear to occur for transient events carrying power in the 150-250 Hz range as reflected in both band-pass filtered LFPs (panels b) and LFP spectrograms (panels c), similar to correct (TP) predictions. One explanation may be that the procedure used to process the data initially either missed SPW-R events with poor signal-to-noise ratio, or that they were rejected manually based on some criteria. The prediction vectors $\hat {y}^{\langle j \rangle }(t)$ approach a value of 1 in some FP cases, implying a high probability of an actual SPW-R event.

For the set of samples resulting in FN predictions shown in Fig. 8, the RippleNet instance may make predictions $\hat {y}(t)$ with magnitudes during the labeled SPW-R events that simply fail to produce a large enough amplitude and/or width for the peak-finding algorithm to detect the event. Here, a reduction of the threshold value for example will reduce FN counts, and increase FP and TP counts (cf. row 1 in Fig. 6b). Other cases resulting in both a FN and FP registration occurs if the predicted event time is outside of the boxcar shapes of the one-hot encoded signal. One such case is occurring in column 2 of Fig. 7.

**Table 6:** TP, FP, TN counts and performance metrics on continuous test set

Model performance summary on continuous test dataset							
model	*TP*	*FP*	*FN*	*FP* + *FN*	*Prec*.	*Recall*	*F*_1_
4	78	85	8	93	0.479	0.907	0.627

### Ripple Detection in Time-Continuous LFP Data

We next pay attention to the litmus test of this project, that is, applications to time continuous LFP recordings of arbitrary durations. We choose the same RippleNet instance as in the previous section, and the 10 minute duration LFP signal of one session of one animal excluded from training or validation data (mouse 4029, session 1, see Table 1). This hold-out data set mimics new recordings unavailable at the time of training the networks. Predicted events within 1s of movement periods are removed from the analysis to suppress FPs resulting from e.g., muscle noise.

By construction, the RippleNet algorithm can, in principle, be run on LFPs of arbitrary duration, even if all training samples are the same duration. We considered two operating modes: Either feeding in the entire LFP sequence as a single sample, or reshaping the LFP sequence into many sequential samples of the same duration. For the latter the predictions made on each sample ($\hat {y}^{\langle j \rangle }(t)$) can be concatenated together to form a continuous signal spanning the duration of the LFP entirely. In practice, 5-fold zero-padding by various amounts and splitting of the LFP signal into samples of duration 0.5 s, running predictions, concatenating predictions, realigning and computing the median output followed by a single, final peak-detection step worked well on the hidden test set.

**Fig. 9:**
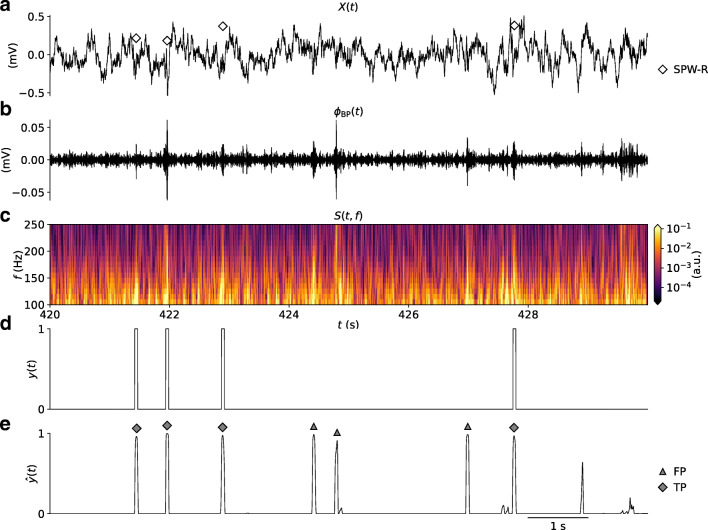
Application of RippleNet on continuous data. **a** 10 s excerpt of input LFP signal *X*(*t*) = *ϕ*(*t*). The *diamonds* marks the times of manually labeled SPW-R events. **b** band-pass filtered LFP *ϕ*_BP_(*t*); **c** Time-frequency resolved spectrogram *S*(*t*,*f*) of the LFP. **d** label array *y*(*t*); **e** prediction $\hat {y}(t)$. The *diamond* and *triangle markers* represents the times of detected TP and FP SPW-R events using the threshold and width parameters that maximize the *F*_1_ score for the model

**Fig. 10:**
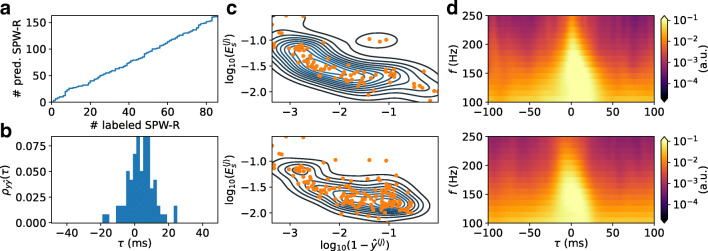
**a** Cumulative counts of predicted SPW-R events as function of labeled SPW-R events. **b** Cross-correlation coefficients between predicted ripple event times and labeled event times as function of time lag *τ* (2ms bin size). **c** Band-pass filtered LFP SPW-R event energy ($E_{\phi _{B}P^{\langle j \rangle }}$) as function of $(1-\hat {y}^{\langle j \rangle })$ of SPW-R events (*orange dots*). The *contour lines* show the bivariate kernel density estimate of the kdeplot method in the Seaborn plotting library. The *top* and *bottom panel* shows labeled and predicted SPW-R events, respectively. **d** Averaged spectrograms for labeled (*top*) and predicted (*bottom*) SPW-R events

For the 10s segment *X*(*t*) shown in Fig. 9a, with corresponding band-pass filtered LFP, spectrogram and one-hot encoded events (Fig. 9b–d), all labeled SPW-R events are found (Fig. 9e). Unsurprisingly, other significant responses with strengths above the peak-finding detection threshold are also found, resulting in an overall larger count of FPs compared to TPs (summarized in Table 6). Based on the above analysis on a validation set with no negative samples that result in an error rate of about one per seven TP SPW-R event, a higher frequency of FP predictions when predictions are made on samples spanning the entire 10 minute session can be expected. The chosen RippleNet instance finds about two times the number of events compared to the number of labeled SPW-R events in the input LFP sequence, see Fig. 10a and Table 6. The cumulative count of predicted events appears linearly dependent on the cumulative count of labeled events. The cross-correlation coefficients $\rho _{y\hat {y}}(\tau )$ between predicted event times and labeled events in the test set (in bins of 2 ms) in Fig. 10b demonstrates a temporally precise prediction of event times, well within the 50 ms boxcar window around each labeled SPW-R event in *y*(*t*) (Fig. 9d).

#### Features of Predicted SPW-R Events are Similar to Labeled Events

**Fig. 11:**
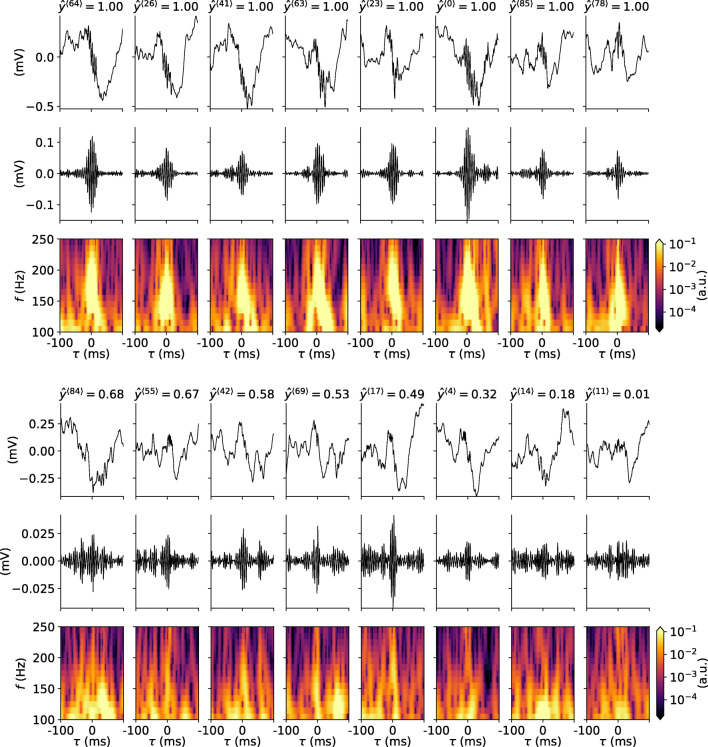
Labeled events (input LFP, band-pass filtered signal, spectrograms) from the hidden test set with highest and lowest RippleNet confidence. Columns in rows 1–3 show eight events which maximized the RippleNet-predicted probabilities ($\hat {y}(t^{\langle j \rangle }) \approx 1$), while rows 4–6 shows labeled events with the lowest predicted event probability

**Fig. 12:**
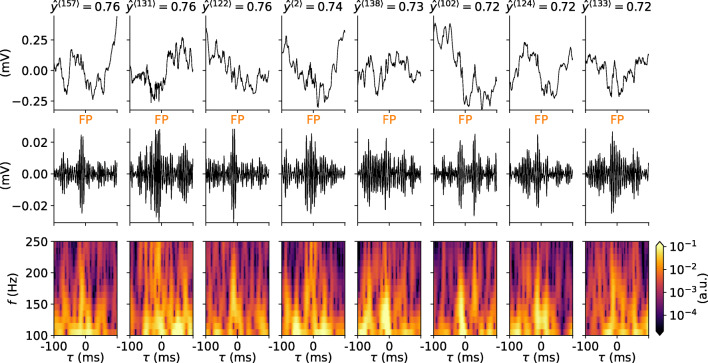
Same as Fig. 11 but for eight FP SPW-R events detected at or above threshold by the RippleNet algorithm

Having established that the chosen RippleNet instance predicts presumed FP events at a high rate relative to TP rate in continuous LFP data, we question if features of predicted events differ from SPW-R events judged by an expert. We first investigate the dependence between predicted SPW-R probability ($\hat {y}^{\langle j \rangle }$) and signal energy in the band-pass filtered LFP $E_{\mathrm {s}}^{\langle j \rangle }$ (14). The RippleNet instance fares well with the labeled events in the hidden test set, with only a handful of FNs but many FPs (summarized in Table 6). The majority of labeled samples result in probabilities $\hat {y}^{\langle j \rangle }$ above the detection threshold 0.7. The eight samples with highest predicted probability are shown in Fig. 11 rows 1–3, and the eight samples with lowest predicted probability in rows 4-6. The RippleNet model instance recognizes SPW-R events with high amplitudes and quite stereotypical appearance both in the band-pass filtered LFP and spectrograms. At the lower end of the scale, SPW-R events show irregular fluctuations at lower amplitudes. The same holds true for the SPW-R events detected above threshold by the RippleNet algorithm (Fig. 12). Detected events have transient activity around 150 Hz in their respective spectrograms, but may otherwise display heterogeneous features.

It thus appears that features of SPW-R events detected by the RippleNet algorithm share features of the manually scored events. In Fig. 10c we plot the signal energy $E_{s}^{\langle j \rangle }$ (14) dependence on probability of *non*-event ($1-\hat {y}^{\langle j \rangle }$) as predicted by the RippleNet instance. In this double-logarithmic plot, the distributions are overlapping, but many more RippleNet-detected events have lower energy and predicted probability. This finding is in agreement with the observed skewed distributions of SPW-R power (see e.g., Csicsvari et al. 1999a). By visual inspection, the averaged spectrogram of labeled and predicted events in Fig. 10d are also very similar in appearance.

## Discussion

In this paper we have introduced the RippleNet algorithm for detecting SPW-R events in time-continuous LFP data as recorded with single- or multi-channel probes in hippocampus CA1. Its development was motivated by high-performance speech recognition systems which utilize deep LSTM based RNNs (Graves et al. 2013; Michalek and Vanek 2018). In the present context, the binary problem of detecting SPW-R events is even simpler than speech recognition which must distinguish between different phonemes making up a spoken language. As such, the SPW-R detection task is analogous to mobile device wake up call detection to commands such as “Hello Siri!” or “OK, Google!” in noisy environments.

We trained two different variants of RippleNet, each instantiated multiple times with different random weights on the same samples from the full set of manually scored data obtained in both mouse and rat CA1. On our validation dataset with mouse and rat data, the best-performing RippleNet instance resulted in 522 TP, 43 FP and 36 FN SWP-R predictions and a combined *F*_1_ score of 0.93 (Table 5). Instances of the non-causal variant of RippleNet utilizing bidirectional LSTMs are found to outperform the causal unidirectional variant during training and validation. On the validation data and with optimized detection threshold settings, unidirectional RippleNets achieved similar TP counts, but with consistently higher error counts than bidirectional variants. For comparison, one well-performing unidirectional RippleNet resulted in 518 TPs, 71 FPs and 38 FNs and *F*_1_ = 0.905.

The fact that the bidirectional variant outperforms the unidirectional variant during training and validation, even if numbers of trainable parameters are larger for the latter case, showcase that also the future context of the input LFP contains information about SPW-R events. Thus, real-time applications of RippleNet, for instance in closed-loop experiments where stimuli is triggered by detected SPW-R events, may be hampered by use of the unidirectional version. In this setting, only use past and present context in order to make a prediction. Thus for offline detection of SPW-R events the better choice is the bidirectional version. As the RippleNet algorithm runs on temporally downsampled LFP signals, a good realtime factor is achievable on short segments, in particular if the computer has GPU accelerating capabilities.

A hidden test dataset was obtained from a single animal with one single session excluded from the training/validation data. This best reflected real-world application to newly obtained LFP recordings in mouse. Features of actual SPW-R events may differ somewhat from those in the training/validation data obtained in different animals and species (Table 1). Test performance (in terms of loss *J*, *MSE*, *Precision*, *Recall*, *F*_1_) can be expected to be reduced compared to results obtained on the train and validation set. Indeed, the resulting counts of 78 TPs, 104 FPs and 8 FNs obtained with the bidirectional RippleNet which performed best on the validation data resulted in poor *Precision* (0.479) but acceptable *Recall* (0.907) and harmonic mean between the two (*F*_1_) of 0.627. We found that application of the RippleNet algorithm results in far more predictions of events with low energy than the conventional detection procedure used to label the test set initially. Superfluous events have similar features to labeled events however.

Given the nature of RNN parameters trained using backpropagation (Hochreiter and Schmidhuber 1997), the RippleNet algorithm may also be sensitive to latent features in the LFP different than high-frequency (around 150 Hz or so) oscillations typically associated with SPW-R events. That raises the question of whether or not conventional SPW-R detection algorithms relying on band-pass filtered LFPs discard useful information contained in other parts of the raw signal. One major caveat to the fact RippleNet algorithm finds most labeled events in the test and validation sets, but also many other positives, imply that the user must still make manual, quite likely subjective, judgements of whether or not detected events are true SPW-R events. As discussed next, results judged by a domain expert can be used to improve the method, along with modifications to the RNN itself.

Additional datasets containing labeled SPW-R events, available from online resources such as https://CRCNS.org(Teeters et al. 2008), can be added as soon as they become available. At present, several CRCNS deposits with CA1 LFPs have been made, but not every dataset comes with labeled SPW-R events. The uploaded datas are mostly obtained in rats using different kinds of electrodes such as laminar probes and tetrodes. Data is also obtained in different brain states such as sleep, anesthetized and awake states. While CA1 SPW-R events may represent underlying brain mechanisms that are highly preserved across species, it is *a priori* unclear if the SPW-R features our algorithm identify in the presently used mouse and rat datasets overlap with those in other data. The SPW-R events may for example have a different distributions of power across frequencies, or typical durations. Thus for the present paper we opted to use only two sources of data, which should each be internally consistent in terms of data quality and methods (species, acquisition hardware, noise levels, data processing steps, label consistency etc.).

With any data and corresponding labels the features any deep learning method may learn is limited if labels are inaccurate. For instance, the rat dataset contained information on SPW-R durations (which we ignored) while the mouse data only contained their occurrence times. More accurate predictions on the existing dataset during training and validation can be achieved by more thorough labeling, perhaps by multiple experts independently.

Synthesizing recordings could also act as a potential supplement to real data. Generative Adverserial Nets (GAN) (Goodfellow et al. 2014) have for instance proven to produce very lifelike data in other domains such as image generation (e.g., Karras et al. 2019). There is an untapped potential to generate virtually unlimited amounts of ‘fake’ LFPs with similar statistics (power spectrum, temporal correlations, etc.) as the real data. A simple SPW-R model based on the superposition of modulated oscillatory events on pink (1/*f*) noise was already proposed by Sethi et al. 2014 (Sethi and Kemere 2014), but pure pink noise can not account for the temporal correlations of real data.

In terms of improving the algorithm itself, RNNs with LSTM layers or Gated Rectified Unit (GRU) layers (Chung et al. 2014) have for some time been considered state-of-the-art in sequence learning (Bai et al. 2018). More recently, alternative architectures such as Temporal Convolutional Networks (TCN), for example WaveNet (van den Oord et al. 2016), also demonstrate capabilities of learning long-term temporal relationships in data. TCN networks were by Bai et al. (2018) shown to outperform LSTM networks on various sequence learning tasks, and should also be evaluated for the SPW-R detection task described throughout this manuscript. The framework developed here around the high-level tensorflow.keras module allows for straightforward comparison between different architectures. This comparison should also include conventional CNNs (LeCun et al. 2015) and variants such as deep residual networks (He et al. 2015) and inception networks (Szegedy et al. 2015; Ismail Fawaz et al. 2019). With the LSTM-based architectures we opted for, one could potentially achieve even better performance by varying hyper parameters for the optimizer (e.g., learning rate), dropout layers (dropout rate), disabling batch-normalizing layers, optimize kernel sizes for the convolutional layers, add additional hidden layers and so forth. While we here did not systematically compare predictions using fundamentally different architectures, we briefly tested multi-layered CNNs, causal TCNs, and replacing LSTM layers with GRU layers, but saw either worse or similar performance on the training and validation data. Similarly, we also tested increasing the layer sizes (and numbers of trainable parameters) and noted longer evaluation times and only slight improvements in accuracy.

As soon as RippleNet has been used to find SPW-R event times in batches of new data, validated SPW-R events can supplement the initial training dataset. Then, the pre-trained RippleNet instance presented here can be trained for more iterations, learn new features and consolidate learned features present in the initial and new samples. With time and several such iterations, an even better performance can be achieved. Another possibility is classification of different kinds of SPW-R events and non-SPW-R (noise) events. We did not yet persuade such classification, as it would require a modification to the final dense output layer to use the so-called Softmax activation function instead of the presently used sigmoid activation function. The output dimensionality and values would then reflect the number of classes and respective probabilities.

In terms of practical usage, pre-trained RippleNet model instances can easily be loaded (with the tf.keras.load_model function in Python), and can be incorporated into Python-based data processing workflows with ease. For this purpose, models may also be converted to the higher-level tf.estimator API. The majority of development and analysis of RippleNet was incorporated using Jupyter notebooks^14^ running on the Google Colaboratory portal^15^ with data file access and synchronization via Google Drive.^16^ RippleNet can be provided as a Cloud service, or as a service running locally on the user’s computer. The latter may facilitate on not having to upload potentially large files but will benefit from a local GPU in order to accelerate compute times. Distribution of RippleNet to end users can be done using containers in Docker,^17^ Kubernetes^18^ or similar. A cloud service however would facilitate on the powerful GPU backends provided via services like Google Cloud^19^ which also has efficient data handling. One option could also be a port of RippleNet to tensorflow.js as the model is already only using keras constructs. The conversion step appears trivial^20^ and could allow execution of RippleNet in html contexts.

The current RippleNet version is set up as a step-by-step workflows in Jupyter notebooks for training, validation and application to continuous data, respectively. While a standalone, interactive RippleNet application with a GUI is certainly possible to develop using cross-platform application tools such as PyQT,^21^ it is presently only considered. A jupyter notebook which allows for user-interactive rejection of detected events (noise events) and storage of accepted events has been implemented, however.

## Outlook

This work constitutes an effort to introduce novel machine learning and deep learning algorithms for the detection of SPW-R events in electrophysiological data. The RippleNet algorithm presented here learns through supervised learning an internal representation of features of SPW-R events. It facilitates a highly non-linear transformation of input LFP signals into output signals that represent the time-varying probabilities of SPW-R events. The approach represents a fundamental change from the typical procedure employed in standard detection workflows relying on hand crafted feature extraction. With access to more training data with labeled events, the method can improve by running more training iterations on new data. RippleNet can reduce the amount of time the experimentalist spend on manual extraction of SPW-R events using heuristic criteria, and allow for a better understanding of features of these events, underlying mechanisms and their role in brain function. We believe that this powerful framework may be adapted to other detection tasks, for instance onset of epileptic seizures, with many potential applications in experimental and clinical settings.

## Footnotes

1 https://www.mathworks.com

2 https://buzsakilab.com/wp/datasets

3 https://www.python.org

4 https://jupyter.org

5 https://numpy.org

6 https://www.scipy.org

7 https://www.h5py.org

8 https://matplotlib.org

9 https://pandas.pydata.org

10 https://www.anaconda.com

11 https://seaborn.pydata.org

12 https://www.tensorflow.org

13 https://colab.research.google.com

14 https://jupyter.org

15 https://colab.research.google.com

16 https://drive.google.com

17 https://www.docker.com

18 https://kubernetes.io

19 cloud.google.com

20 https://www.tensorflow.org/js/tutorials/conversion/import_keras

21 https://www.riverbankcomputing.com/static/Docs/PyQt5
